# Molecular Characterization of Bovine SMO Gene and Effects of Its Genetic Variations on Body Size Traits in Qinchuan Cattle (*Bos taurus*)

**DOI:** 10.3390/ijms160816966

**Published:** 2015-07-27

**Authors:** Ya-Ran Zhang, Lin-Sheng Gui, Yao-Kun Li, Bi-Jie Jiang, Hong-Cheng Wang, Ying-Ying Zhang, Lin-Sen Zan

**Affiliations:** 1 College of Animal Science and Technology, Northwest A&F University, Yangling 712100, Shaanxi, China; E-Mails: zhang_ya_ran@126.com (Y.-R.Z.); guilinsheng1234@163.com (L.-S.G.); liyaokun1986@gmail.com (Y.-K.L.); jiangbijie1001@163.com (B.-J.J.); besthongcheng@163.com (H.-C.W.); zyy686868@163.com (Y.-Y.Z.); 2 National Beef Cattle Improvement Center of Northwest A&F University, Yangling 712100, Shaanxi, China

**Keywords:** SMO gene, molecular characterization, body size traits, Qinchuan cattle

## Abstract

Smoothened (Smo)-mediated Hedgehog (Hh) signaling pathway governs the patterning, morphogenesis and growth of many different regions within animal body plans. This study evaluated the effects of genetic variations of the bovine SMO gene on economically important body size traits in Chinese Qinchuan cattle. Altogether, eight single nucleotide polymorphisms (SNPs: 1–8) were identified and genotyped via direct sequencing covering most of the coding region and 3ʹUTR of the bovine SMO gene. Both the p.698Ser.>Ser. synonymous mutation resulted from SNP1 and the p.700Ser.>Pro. non-synonymous mutation caused by SNP2 mapped to the intracellular C-terminal tail of bovine Smo protein; the other six SNPs were non-coding variants located in the 3ʹUTR. The linkage disequilibrium was analyzed, and five haplotypes were discovered in 520 Qinchuan cattle. Association analyses showed that SNP2, SNP3/5, SNP4 and SNP6/7 were significantly associated with some body size traits (*p* < 0.05) except SNP1/8 (*p* > 0.05). Meanwhile, cattle with wild-type combined haplotype Hap1/Hap1 had significantly (*p* < 0.05) greater body length than those with Hap2/Hap2. Our results indicate that variations in the SMO gene could affect body size traits of Qinchuan cattle, and the wild-type haplotype Hap1 together with the wild-type alleles of these detected SNPs in the SMO gene could be used to breed cattle with superior body size traits. Therefore, our results could be helpful for marker-assisted selection in beef cattle breeding programs.

## 1. Introduction

Smoothened (Smo)-mediated Hedgehog (Hh) signaling pathway is a critical regulator of cell growth and patterning during embryonic development, and also is involved in stem cell renewal and tissue homeostasis in adult animals [1,2,3]. Perturbations in the pathway are related to birth defects and various cancers [4]. Smo, a seven-transmembrane protein, belongs to the Frizzled (FzD) class of G-protein-coupled receptor (GPCR) superfamily and serves as the obligatory signal transducer of the Hh pathway [5,6,7]. Specifically, Smo transforms the extracellular Hh protein signal into an intracellular glima-associated transcription factors (Gli1-3) protein signal, thereby activating intranuclear target genes [8,9]. As a core element of the Hh pathway, SMO gene is conserved from flies to humans [1], and enormous progress has been made in revealing not only its molecular structure and functional mechanism but also its cellular and developmental functions. Functional studies in model animals have shown that SMO gene participates in both osteogenesis and myogenesis through Hh pathway.

In mice, the forced expression of a constitutively actived SMO allele promoted chondrocyte proliferation, while chondrocyte-specific SMO knockout mice showed a marked decrease in chondrocyte proliferation and developed shorter long bones than wild-type littermates [10]. Similarly, the removal of SMO from perichondrial cells led to bone collar defects and abolished development of primary spongiosa. Meanwhile, in chimeric mice, cells genetically deficient in SMO exhibited a cell-autonomous defect in osteoblast differentiation in bone collar and primary spongiosa [11]. In zebrafish, inactive Smo mutant embryos displayed phenotypic defects, including abnormalities in body size, cartilage, pectoral fins, central nervous system [12]. Compared to wild-type zebrafish embryos, Smo mutant embryos lacked slow muscle, and their fast muscle fibers were disorganized [13]. A recent study reported that the fine coordination of Smo activity by the miR-30 family controlled the specification and differentiation of distinct muscle cell types of zebrafish embryos [14]. Collectively, these findings confirm SMO contributes to the development and growth of bone and muscle, suggesting that SMO is an attractive candidate gene for the selection of growth-related traits in livestock.

Little study on the bovine SMO gene has been performed, since most research has focused on model animals and human. Based on its role in osteogenesis and myogenesis as demonstrated in mice and zebrafish, we proposed the hypothesis that variations of the SMO gene affected body size traits in cattle. Here, we do research on Qinchuan cattle, for as a famous indigenous cattle breed in China, its growth rate and underdeveloped hind hip need to be improved to be comparable to imported beef cattle breeds [15]. Molecular characterization of the bovine SMO was analyzed using bioinformatics. Screening of genetic variations was performed by direct sequencing. Then the genetic associations with body size traits in Qinchuan cattle were examined. Our results are potentially beneficial for further research in enhancing the economic traits of beef cattle.

## 2. Results and Discussion

### 2.1. Sequence Homology, Inferred Phylogenetic Tree and Sequence Alignments

**Figure 1:**
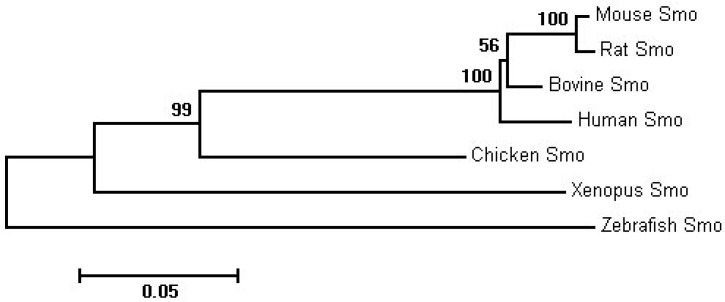
Phylogenetic tree for amino acid sequences of the SMO gene in seven species with bootstrap confidence values at the branch nodes. Branch lengths indicated the evolutionary distances.

SMO gene maps to bovine chromosome 4, consists of 12 exons divided by 11 introns and 3ʹUTR, and encodes 780 amino acids. BLAST analysis revealed that the amino acid sequence of the bovine SMO (NP_001179149.1) shared high identity with other vertebrates. The percent identity with human (NP_005622.1), rat (NP_036939.1), mouse (NP_795970.3), chicken (XP_414970.4), zebrafish (NP_571102.1) and xenopus (NP_001128704.1) were 94%, 93%, 92%, 79%, 71% and 70%, respectively. The relatively high identity among mammalians (92%–94%) suggested that the SMO gene was more conserved within this group. The phylogenetic tree (Figure 1) intuitively showed the relationship between bovine SMO and the potential evolutionary process; bovine, human, mouse and rat SMO fell into one evolutionarily related group, while chicken, xenopus and zebrafish SMO were dispersed at more distant branches.

**Figure 2:**
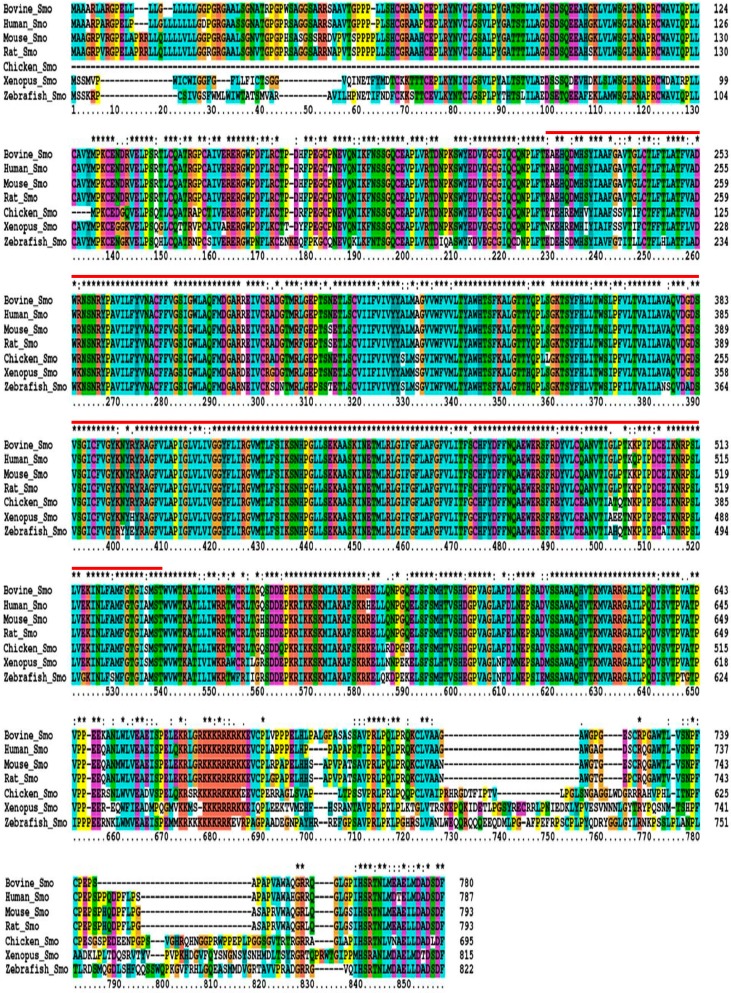
Aligned multiple amino acid sequences of the SMO gene in seven species. To make the assessment of alignment much easier, amino acid residues with different chemical properties are differentiated with different background colors. Asterisks denote positions that have been fully conserved; the “:” and “.” characters indicate positions where conservative and semi-conserved amino acid mutations have happened, respectively. And the red line highlights the inferred transmembrane domain.

Research on model animals and human has revealed that Smo protein consists of three domains: the extracellular N-terminal domain, a seven heptahelical transmembrane domain, and the intracellular C-terminal tail [16]. Using the already known information of other species, to better understand the structure of bovine Smo protein, we performed multiple alignments of the amino acid sequences among the above mentioned species. Figure 2 showed the aligned Smo amino acid sequences; asterisks denoted the fully conserved amino acid residues. Based on the recently determined three-dimensional structure of human Smo (hSmo) [16], analysis indicated that in the seven species, the most highly conserved region covered the seven heptahelical transmembrane domain (residues 224–534 of hSmo), in accordance with the common feature in all GPCRs [17]. The regions comprising the transmembrane domain were highlighted with a red line. The upstream and downstream the transmembrane domain were the extracellular N-terminal and intracellular C-terminal domains, respectively.

### 2.2. Analysis of Sequence Variations in Qinchuan Cattle SMO Gene

**Figure 3:**
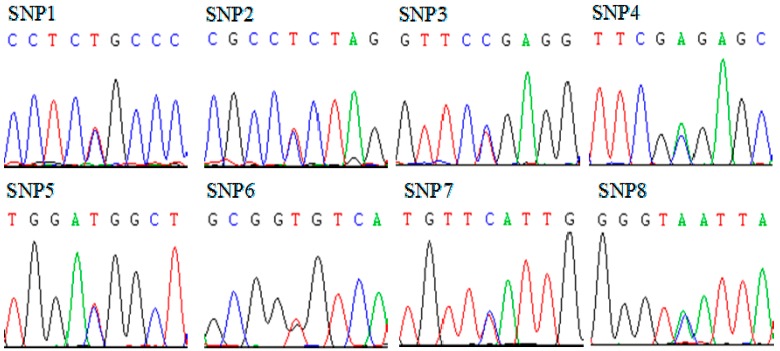
The sequencing maps of the eight detected single nucleotide polymorphisms (SNPs) within the bovine SMO gene. SNP1: g.22935C>T; SNP2: g.22939T>C; SNP3: g.23232C>T; SNP4: g.23283C>A; SNP5: g.23329C>T; SNP6: g.23458T>G; SNP7: g.23633T>C; SNP8: g.23641C>A.

We amplified and sequenced most of the coding region and 3ʹUTR of the SMO gene. Compared with the SMO gene reference sequence (GenBank accession No. AC_000161), a total of eight SNPs (Figure 3), including two coding variants (SNP1: g.22935C>T, SNP2: g.22939T>C) in exon 12 and six non-coding variants (SNP3: g.23232C>T, SNP4: g.23283C>A, SNP5: g.23329C>T, SNP6: g.23458T>G, SNP7: g.23633T>C, SNP8: g.23641C>A) in the 3ʹUTR, were identified in 520 Qinchuan cattle. SNP1 (g.22935C>T) resulted in a p.698Ser.>Ser. synonymous mutation and SNP2 (g.22939T>C) led to a p.700Ser.>Pro. missense mutation. According to the above aligned Smo amino acid sequences (Figure 2), both the p.698Ser.>Ser. synonymous mutation and the p.700Ser.>Pro. non-synonymous mutation were located in the intracellular C-terminal tail of bovine Smo. In addition, compared with the public SNP information of the bovine SMO gene provided by NCBI, SNP1 and SNP8 were identified as two novel variations and will be submitted into the SNP data bank.

All eight SNPs were successfully genotyped by PCR-direct sequencing. The detailed SNP information for each cattle was provided in Table S1. The two novel SNPs (SNP1 and SNP8) exhibited two different genotypes: wild-type homozygote and heterozygote; whereas, the others displayed three kinds of genotypes: wild-type homozygote, heterozygote and mutant homozygote (Table 1).

Table 1 also contained the results of genotypic and allelic frequencies, genetic diversity parameters (He, Ne and PIC) and Hardy-Weinberg equilibrium. The wild-type alleles of all eight SNP loci were predominant in the studied population. Results of χ^2^ test indicated that genotype distributions of the eight mutations were all in Hardy-Weinberg equilibrium (χ^2^ < χ^2^_0.05_). The PIC value (if PIC < 0.25, 0.25 < PIC < 0.5, or PIC > 0.5, low, medium or high polymorphism, respectively [18]) manifested that the two novel SNPs were in low polymorphism, while the rest were located on medium genetic diversity level in the studied population.

**Table 1:** Genotypic, allelic frequencies and genetic diversity of eight SNP loci within the SMO gene in Qinchuan cattle populations.

Loci	Genotypic Frequencies			Total	Allelic Frequencies		χ^2^ (HWE)	PIC	He	Ne
	Wild Type	Hybrid Subtype	Mutant Type		Wild Type	Mutant Type				
SNP1/8	CC/CC	CT/CA	TT/AA	520	C/C	T/A	0.5584	0.0596	0.0614	1.0655
	0.9365	0.0635	0		0.9683	0.0317				
SNP2	TT	TC	CC	520	T	C	1.0186	0.3708	0.4917	1.9673
	0.3077	0.5135	0.1788		0.5644	0.4356				
SNP3/5	CC/CC	CT/CT	TT/TT	520	C/C	T/T	0.0292	0.2920	0.3550	1.5505
	0.5904	0.3577	0.0519		0.7692	0.2308				
SNP4	CC	CA	AA	520	C	A	0.2585	0.3496	0.4515	1.8231
	0.4250	0.4615	0.1135		0.6558	0.3442				
SNP6/7	TT/TT	TG/TC	GG/CC	520	T/T	G/C	0.1634	0.3527	0.4573	1.8426
	0.4135	0.4654	0.1212		0.6462	0.3538				

HWE, Hardy-Weinberg equilibrium; χ^2^_0.05_ = 5.991; χ^2^_0.01_ = 9.210.

### 2.3. Linkage Disequilibrium and Haplotype Analysis

Table 2 illustrated the results of linkage disequilibrium (LD). According to the *r*^2^-value, a pairwise measure of LD, SNP1 and SNP8 loci were in perfect LD (*r*^2^ = 1), so were SNP3 and SNP5 (*r*^2^ = 1), and SNP6 and SNP7 (*r*^2^ = 1). *r*^2^ = 1 indicates that, during the history of the sample, two SNPs have not been separated by recombination, and observations at one SNP provide complete information about the other SNP [19]. Thus, SNP1 and SNP8 loci were abbreviated as a single SNP1/8 locus, SNP3 and SNP5 as SNP3/5, and SNP6 and SNP7 as SNP6/7. In addition, SNP2, SNP3/5, SNP4 and SNP6/7 loci exhibited strong LD (*r*^2^ > 0.33) with each other. This may be a result of selection which can result in nonrandom associations among SNPs and thus elevate levels of LD in a gene; especially selection, during domestication, aiming at alleles of a gene can significantly elevate levels of LD in a given region [20,21].

**Table 2:** Estimated values of linkage disequilibrium analysis for eight detected SNPs.

*r*^2^	SNP2	SNP3	SNP4	SNP5	SNP6	SNP7	SNP8
SNP1	0.042	0.008	0.062	0.008	0.060	0.060	1.000
SNP2		0.389	0.680	0.389	0.710	0.710	0.042
SNP3			0.571	1.000	0.548	0.548	0.008
SNP4				0.571	0.385	0.385	0.062
SNP5					0.548	0.548	0.008
SNP6						1.000	0.060
SNP7							0.060

If *r*^2^ = 1, SNP pairs are in perfect linkage disequilibrium; If *r*^2^ > 0.33, SNP pairs are in strong linkage disequilibrium.

Subsequently, a total of five haplotypes were identified (Table 3). Theoretically, the number of the inferred haplotypes should be 256 (2^8^), but both the perfect and strong LDs between SNP marker pairs notably decreased the number. Obviously, Hap1 (-CTCCCTTC-), the combination of wild-type alleles, was the most frequent (0.558), followed by Hap2 (0.234), Hap3 (0.092), Hap4 (0.085), and lastly the combination of mutant-type alleles Hap5 (-TCCACGCA-) (0.031). The high-frequency haplotypes might have been in the population for a long time and more adaptive to the environment or selection [22,23]. Consequently, new mutants are more likely to originate from the common haplotypes, meaning that rarer variants are more closely related to the common haplotypes and represent recent mutations [24].

**Table 3:** Information of haplotypes of the SMO gene in Qinchuan cattle population.

Haplotype	SNPs								Frequency
	1	2	3	4	5	6	7	8	
Hap1	C	T	C	C	C	T	T	C	0.564
Hap2	C	C	T	A	T	G	C	C	0.231
Hap3	C	C	C	C	C	G	C	C	0.091
Hap4	C	C	C	A	C	T	T	C	0.082
Hap5	T	C	C	A	C	G	C	A	0.032

### 2.4. Effects of Single Marker on Body Size Traits

Association analysis between the single SNP markers and body size traits were performed (Table 4). At the SNP1/8 locus, there were no significant effects on body size traits (*p* > 0.05) (data not shown).

At the SNP2 locus, animals with wild-type genotype TT had significantly greater body length (*p* = 0.005), wither height (*p* = 0.011), hip height (*p* = 0.022), hip width (*p* = 0.004) and heart girth (*p* = 0.018) than those with mutant genotype CC; significant difference was also found between genotype TC and CC for hip width (*p* = 0.015). As mentioned above, SNP2 led to the p.700Ser.>Pro. non-synonymous mutation in the intracellular C-terminal tail of bovine Smo. Intriguingly, in common with other GPCRs, phosphorylation of Smo regulates the switch between on/off signaling states [6]; or rather the activation of Smo is triggered by hyperphosphorylating Ser/Thr residues within C-terminal cytoplasmic tail during Hh signaling [25,26,27,28]. Substitutions of phosphorylation sites with un-phosphorylatable residues render Smo inactive and diminish Hh signal activity, whereas phosphor-mimetic Smo variants display overactive Hh signal [25,29,30]. Moreover, with the increase in the number of phosphor-mimetic mutations in its C-terminal tail, Smo activity exhibits a progressive elevation. [30]. Herein, we deduced that individuals with mutant allele SNP2-C showed significantly lower body size traits than those with wild-type allele SNP2-T (*p* < 0.05) might be caused by the attenuation of Smo activation via the substitution of Ser. residue with un-phosphorylatable residue Pro. Subsequently, the attenuation might undergo cascade amplification, consequently influencing the transcription of downstream target genes.

**Table 4:** Associations between different genotypes of SNPs detected in SMO and body size traits in Qinchuan cattle.

Loci	Genotypes	Body Size Traits (Mean ± SE)						
		BL (cm)	WH (cm)	HH (cm)	RL (cm)	HW (cm)	CD (cm)	HG (cm)
SNP 2	TT (160)	135.422 ± 0.934 ^a^	121.937 ± 0.654 ^a^	124.422 ± 0.562 ^a^	42.263 ± 0.349	39.025 ± 0.451 ^a^	59.156 ± 0.539	164.131 ± 1.397 ^a^
	TC (267)	133.333 ± 0.723	120.936 ± 0.506	123.414 ± 0.435	42.049 ± 0.270	38.577 ± 0.349 ^a^	58.470 ± 0.417	162.534 ± 1.067
	CC (93)	130.565 ± 1.225 ^b^	118.790 ± 0.858 ^b^	121.925 ± 0.738 ^b^	41.215 ± 0.458	36.645 ± 0.592 ^b^	57.301 ± 0.112	157.871 ± 1.808 ^b^
	*p*-value ^1^	0.005	0.011	0.022	0.208	0.004/0.015	0.081	0.018
SNP3/5	CC/CC (307)	134.718 ± 0.673 ^a^	121.406 ± 0.473 ^a^	123.785 ± 0.407	42.231 ± 0.252	38.827 ± 0.327 ^a^	58.866 ± 0.389	163.383 ± 0.995 ^a^
	CT/CT (186)	132.239 ± 0.865	120.470 ± 0.608	123.312 ± 0.523	41.677 ± 0.324	37.995 ± 0.420	58.081 ± 0.500	161.409 ± 1.279
	TT/TT (27)	127.963 ± 2.271 ^b^	117.352 ± 1.595 ^b^	120.741 ± 1.372	40.926 ± 0.850	35.741 ± 1.101 ^b^	56.685 ± 1.312	154.037 ± 3.356 ^b^
	*p*-value ^1^	0.014	0.046	0.102	0.424	0.022	0.335	0.024
SNP4	CC (221)	135.262 ± 0.794 ^a^	121.776 ± 0.558 ^a^	124.122 ± 0.480	42.235 ± 0.297	38.905 ± 0.385 ^a^	59.102 ± 0.458	163.833 ± 1.176
	CA (240)	132.650 ± 0.762	120.512 ± 0.535	123.198 ± 0.460	41.854 ± 0.285	38.292 ± 0.370	58.160 ± 0.440	161.723 ± 1.128
	AA (59)	130.186 ± 1.537 ^b^	118.847 ± 1.079 ^b^	122.025 ± 0.928	41.407 ± 0.576	36.678 ± 0.746 ^b^	57.381 ± 0.887	157.949 ± 2.275
	*p*-value ^1^	0.010	0.049	0.136	0.605	0.025	0.257	0.066
SNP6/7	TT/TT (215)	134.935 ± 0.808 ^a^	121.800 ± 0.565	124.277 ± 0.486	42.326 ± 0.301	38.972 ± 0.390 ^a^	58.988 ± 0.465	163.579 ± 1.189 ^a^
	TG/TC (242)	132.963 ± 0.761	120.512 ± 0.533	123.054 ± 0.458	41.864 ± 0.284	38.326 ± 0.368	58.227 ± 0.439	162.388 ± 1.121
	GG/CC (63)	130.508 ± 1.492 ^b^	118.992 ± 1.045	122.214 ± 0.898	41.127 ± 0.556	36.476 ± 0.720 ^b^	57.651 ± 0.860	156.698 ± 2.197 ^b^
	*p*-value ^1^	0.028	0.055	0.132	0.176	0.007	0.516	0.018

^a,b^ Means with different superscripts are significantly different (*p* < 0.05); ^1^ *p-*values after modified Bonferroni correction for trait-wise multiple tests.

At the SNP3/5 locus, animals with wild-type genotype CC/CC showed significantly larger body length (*p* = 0.014), wither height (*p* = 0.046), hip width (*p* = 0.022) and heart girth (*p* = 0.024) compared to those with mutant genotype TT/TT. At the SNP4 locus, individuals with genotype CC demonstrated higher mean values for body length (*p* = 0.010), wither height (*p* = 0.049) and hip width (*p* = 0.025) than those with genotype AA. At the SNP6/7 locus, the body length (*p* = 0.028), hip width (*p* = 0.007) and heart girth (*p* = 0.018) of animals with wild-type genotype TT/TT were significantly larger than those of animals with mutant genotype GG/CC. Although these SNPs identified in the 3ʹUTR were non-coding DNA variants, there is growing indication that 3ʹUTR variants actively participate in modifying gene expression patterns. A G > A transition in the 3ʹUTR of the GDF8 gene created microRNA target sites for mir1 and mir206, consequently promoting the muscular hypertrophy phenotype in Texel sheep [31]. Additionally, an A > G substitution in the 3ʹUTR of the pig PPARA gene associated with adipose tissue accumulation was found located near the putative target sequence for mir224 and potentially increased the mir224 binding to the PPARA, thus reducing PPARA transcript level [32]. Accordingly, we hypothesized that variants in the 3ʹUTR of the bovine SMO gene might work in a manner parallel to SNPs in the 3ʹUTR of the GDF8 or PPARA gene, such as creating microRNA binding sites or possibly influencing the combination of the potential microRNAs with the SMO gene, thereby influencing the transcription level of SMO.

Collectively, wild-type individuals had larger body size traits in our Qinchuan cattle population, and wild-type alleles (SNP2-T, SNP3/5-C/C, SNP4-C, and SNP6/7-T/T) appeared to be beneficial for improving body size traits in cattle breeding programs.

### 2.5. Effects of Haplotype Combinations on Body Size Traits

Haplotype combinations may provide greater power than a single marker for genetic disease and trait associations [33]. Consequently, we analyzed the haplotype combinations of eight SNPs, and a total of 14 diplotypes (combined genotypes or haplotypes) were identified. We selected five diplotypes for association analysis; those with frequency far lower than 0.05 were not chosen. Individuals with wild-type diplotype Hap1/Hap1 (CC-TT-CC-CC-CC-TT-TT-CC) displayed significantly greater body length (*p* = 0.031) than those with Hap2/Hap2 (CC-CC-TT-AA-TT-GG-CC-CC) (Table 5), which suggested that diplotype Hap1/Hap1 could be used as a molecular marker in selection of preferable body size traits in cattle. Likewise, the results were in agreement with the conclusion of the effect of one SNP, the wild-type alleles (SNP2-T, SNP3/5-C/C, SNP4-C, and SNP6/7-T/T) related with greater body size traits. On the other hand, statistical analyses implied that mutations of these detected loci within SMO might lead to a decrease in body size traits.

**Table 5:** Associations of daplotypes with body size traits in Qinchuan cattle.

Diplotype	Body Size Traits (Mean ± SE)						
	BL (cm)	WH (cm)	HH (cm)	RL (cm)	HW (cm)	CD (cm)	HG (cm)
Hap1/Hap1 (160)	135.422 ± 0.952 ^a^	121.938 ± 0.668	124.422 ± 0.578	42.263 ± 0.354	39.025 ± 0.456	59.156 ± 0.547	164.131 ± 1.375
Hap1/Hap2 (139)	132.327 ± 1.022	120.478 ± 0.716	123.183 ± 0.620	41.705 ± 0.380	38.252 ± 0.489	58.119 ± 0.587	161.935 ± 1.476
Hap1/Hap3 (59)	135.246 ± 1.568	121.619 ± 1.099	123.636 ± 0.951	42.339 ± 0.583	38.898 ± 0.750	59.237 ± 0.900	163.525 ± 2.265
Hap1/Hap4 (53)	133.387 ± 1.655	121.491 ± 1.160	123.943 ± 1.003	42.509 ± 0.615	38.679 ± 0.792	58.387 ± 0.950	161.519 ± 2.390
Hap2/Hap2 (27)	127.963 ± 2.318 ^b^	117.352 ± 1.625	120.741 ± 1.406	40.926 ± 0.862	35.741 ± 1.109	56.685 ± 1.331	154.037 ± 3.348
*p*-value ^1^	0.031	0.094	0.158	1.000	0.064	0.866	0.055

^a,b^ Means with different superscripts are significantly different (*p* < 0.05); ^1^ *p-*values after modified Bonferroni correction for trait-wise multiple tests.

Marker-assisted selection (MAS) based on genetic variation is more effective and powerful than traditional selection methodologies for genetically improving livestock economic traits, for example, growth traits, milk traits, reproduction traits, meat quality traits [34]. It is well established that genes involved in Hh signaling, including IHH, SHH, DHH, PTCH1, SMO and GLI1-3, play crucial roles in the growth, patterning and morphogenesis of various animal tissues [1,8,9]. Genome-wide association analysis in human has documented that variants within genes in Hh signaling are associated with adult height [35]. Studies in mice and zebrafish have demonstrated that SMO contributes to osteogenesis and myogenesis, consequently influencing the development and growth of bone and muscle [10,11,12,13,14]. These findings suggest that the SMO gene, as one mediating Hh signaling, could be a potential candidate gene related to animal body size traits. Our results showed that some variations within SMO were associated with body size traits in Qinchuan cattle, making it useful as a molecular marker in the MAS program for beef cattle.

## 3. Experimental Section

### 3.1. Animal Source, Data Collection and Genomic DNA Preparation

520 adult animals (18–24 months old; unrelated for at least three generations) were selected from the following farms: Fineness Breeding Center of Qinchuan Cattle (Yangling, Shaanxi, China), Reserved Farm of Qinchuan Cattle (Fufeng, Shaanxi, China) and Qinchuan Cattle Farm (Qian County, Shaanxi, China). Seven body measurement traits (body length (BL),wither height (WH), hip height (HH), rump length (RL), hip width (HW), chest depth (CD), heart girth (HG)) were measured for statistical analysis as Gilbert *et al.* [36] described. Meanwhile, blood samples were collected from the jugular vein and immediately treated with 2.0% heparin. Genomic DNA samples were extracted from blood using the standard phenol-chloroform protocol [37].

### 3.2. Bioinformatic Study

The amino acid sequences of the SMO gene for different species (*Bos taurus*, *Homo sapiens*, *Rattus norvegicus*, *Mus musculus*, *Gallus gallus*, *Xenopus laevis* and *Danio rerio*) were acquired using BLAST provided by NCBI. A phylogenetic tree for SMO was constructed using MEGA6.06 software. Multiple sequence alignment for orthologous Smo proteins was performed using Clustalx2.0 software.

### 3.3. Sequence Variant Detection

Primers (Table S2) used to amplify the bovine SMO gene were designed based on the published nucleotide sequence (GenBank accession No. AC_000161). Polymerase chain reaction (PCR) was conducted in 30 µL reaction mixtures, containing 10 pM of each primer, 50 ng templates DNA, 15 µL 2× Reaction Mix (500 M dNTP, 20 mM Tris-HCl (pH 8.3), 100 mM KCl, 3 mM MgCl_2_, other stabilizer and enhancer), and 0.3U Golden DNA polymerase (Tiangen Biotech, Beijing, China). The PCR was performed in a thermal cycler (Eppendorf, Germany) with the following procedure: initial denaturing at 95 °C for 5 min, followed by 35 cycles of 30 s at 94 °C, 30 s at the optimum annealing temperature (Table S1), 72 °C for 1 min, and ended with a final elongation of 10 min at 72 °C. PCR products were detected by electrophoresis on 1.5% (*w*/*v*) agarose gel (containing 200 ng/mL ethidium bromide) and purified by Axygen kits (MBI Fermentas, Amherst, NY, USA), and then sequenced in both directions in an ABI PRIZM377 DNA analyzer (Perkin-Elmer, Waltham, MA, USA). Sequence maps were imported into SeqMan of DNASTAR software (version 7.1) and analyzed to search for variations.

### 3.4. Genotyping

Primers (Table S2) were redesigned for genotyping eight detected SNPs via direct sequencing. PCR conditions, sequencing and sequence analysis were as described above.

### 3.5. Statistical Analyses

Genotype frequencies were calculated by direct counting. Allele frequencies, Hardy-Weinberg equilibrium (HWE), heterozygosity (He), and effective allele numbers (Ne) were analyzed based on genotype frequencies as Nei and Rychoudhury [38] described; polymorphism information content (PIC) was obtained via Botstein’s methods [18]. Linkage disequilibrium between all pairs of biallelic loci, as well as haplotypes, was analyzed using the Partition-Ligation Combination-Subdivision EM (PL-CSEM) algorithm of SHEsis software [39,40].

The associations between single SNP marker and body measurement traits were analyzed with SPSS software (version .19.0) using the general linear models (GLM) procedure and the following model:

(1) Y_ijklm_ = μ + G_i_ + A_j_ +F_k_ +S_l_ + S_m_ + ε_ijklm_

where Y_ijklm_ = trait measured on each individual; μ = overall mean; G_i_ = fixed effect of the ith genotype; A_j_ = fixed effect of the jth age; F_k_ = fixed effect of the kth farm; S_l_ = fixed effect of the lth sex; S_m_ = fixed effect of the mth sire; and ε_ijklm_ = random error.

For the association analysis between combined genotypes and the body size traits, the statistical model was similar to the model 1 with a slight modification, which was that Gi was the fixed effect associated with the ith combined genotypes. Moreover, to obtain more robust results, all the p values of the statistical results were corrected by Bonferroni correction which was used to account for multiple tests.

## 4. Conclusions

In summary, we analyzed the molecular characterization of the bovine SMO together with the SMO of several other species, and identified eight SNPs and five haplotypes in the coding region and 3ʹUTR of SMO in 520 Qinchuan cattle. Genotyping and association analyses demonstrated that wild-type alleles of some detected SNPs appeared to be more beneficial for selecting cattle with superior body size traits, and could be used as molecular markers for the improvement of Qinchuan cattle and perhaps other breedtypes that would benefit from marker-assisted selection for increased body size. Nevertheless, these results should be considered as preliminary, and further studies should be conducted to validate observed associations in a broad variety of cattle breeds. Further studies also should explore the molecular mechanisms responsible for these SNPs in the SMO gene associated with the variations in body size traits.

## Supplementary Materials

Supplementary materials can be found at http://www.mdpi.com/1422-0067/16/08/16966/s1.

## References

[1] Ingham P.W., McMahon A.P. (2001). Hedgehog signaling in animal development: Paradigms and principles. Genes Dev..

[2] Beachy P.A., Karhadkar S.S., Berman D.M. (2004). Tissue repair and stem cell renewal in carcinogenesis. Nature.

[3] Jiang J., Hui C.C. (2008). Hedgehog signaling in development and cancer. Dev. Cell.

[4] Sharpe H.J., Wang W., Hannoush R.N., de Sauvage F.J. (2015). Regulation of the oncoprotein smoothened by small molecules. Nat. Chem. Biol..

[5] Alcedo J., Ayzenzon M., von Ohlen T., Noll M., Hooper J.M. (1996). The Drosophila smoothened gene encodes a seven-pass membrane protein, a putative receptor for the hedgehog signal. Cell.

[6] Ayers K.L., Therond P.P. (2010). Evaluating smoothened as a G-protein-coupled receptor for Hedgehog signaling. Trends Cell Biol..

[7] Wang C., Wu H., Katritch V., Han G.W., Huang X.P., Liu W., Siu F.Y., Roth B.L., Cherezov V., Stevens R.C. (2013). Structure of the human smoothened receptor bound to an antitumour agent. Nature.

[8] McMahon A.P., Ingham P.W., Tabin C.J. (2003). Developmental roles and clinical significance of hedgehog signaling. Curr. Top. Dev. Biol..

[9] Robbins D.J., Fei D.L., Riobo N.A. (2012). The Hedgehog signal transduction network. Sci. Signal..

[10] Long F., Zhang X.M., Karp S., Yang Y., McMahon A.P. (2001). Genetic manipulation of hedgehog signaling in the endochondral skeleton reveals a direct role in the regulation of chondrocyte proliferation. Development.

[11] Long F., Chung U.I., Ohba S., McMahon J., Kronenberg H.M., McMahon A.P. (2004). Ihh signaling is directly required for the osteoblast lineage in the endochondral skeleton. Development.

[12] Chen W., Burgess S., Hopkins N. (2001). Analysis of the zebrafish smoothened mutant reveals conserved and divergent functions of hedgehog activity. Development.

[13] Henry C.A., Amacher S.L. (2004). Zebrafish slow muscle cell migration induces a wave of fast muscle morphogenesis. Dev. Cell.

[14] Ketley A., Warren A., Holmes E., Gering M., Aboobaker A.A., Brook J.D. (2013). The miR-30 MicroRNA family targets smoothened to regulate Hedgehog signalling in Zebrafish early muscle development. PLoS ONE.

[15] Adoligbe C., Zan L., Farougou S., Wang H., Ujjan J.A. (2012). Bovine GDF10 gene polymorphism analysis and its association with body measurement traits in Chinese indigenous cattle. Mol. Biol. Rep..

[16] Rognan D., Mus-Veteau I. (2014). Three-Dimensional structure of the smoothened receptor: Implications for drug discovery. Top. Med. Chem..

[17] Venkatakrishnan A.J., Deupi X., Lebon G., Tate C.G., Schertler G.F., Babu M.M. (2013). Molecular signatures of G-protein-coupled receptors. Nature.

[18] Botstein D., White R.L., Skolnick M., Davis R.W. (1980). Construction of a genetic linkage map in man using restriction fragment length polymorphisms. Am. J. Hum. Genet..

[19] Ardlie K.G., Kruglyak L., Seielstad M. (2002). Patterns of linkage disequilibrium in the human genome. Genetics.

[20] Saunders M.A., Slatkin M., Garner C., Hammer M.F., Nachman M.W. (2005). The extent of linkage disequilibrium caused by selection on G6PD in humans. Genetics.

[21] Clark R.M., Linton E., Messing J., Doebley J.F. (2004). Pattern of diversity in the genomic region near the maize domestication gene tb1. Proc. Natl. Acad. Sci. USA.

[22] Huang Y.Z., Wang K.Y., He H., Shen Q.W., Lei C.Z., Lan X.Y., Zhang C.L., Chen H. (2013). Haplotype distribution in the GLI3 gene and their associations with growth traits in cattle. Gene.

[23] Wang G., Zhang S., Wei S., Zhang Y., Li Y., Fu C., Zhao C., Zan L. (2014). Novel polymorphisms of SIX4 gene and their association with body measurement traits in Qinchuan cattle. Gene.

[24] Posada D., Crandall K.A. (2001). Intraspecific gene genealogies: Trees grafting into networks. Trends Ecol. Evol..

[25] Apionishev S., Katanayeva N.M., Marks S.A., Kalderon D., Tomlinson A. (2005). Drosophila Smoothened phosphorylation sites essential for Hedgehog signal transduction. Nat. Cell Biol..

[26] Chen Y., Li S., Tong C., Zhao Y., Wang B., Liu Y., Jia J., Jiang J. (2010). G protein-coupled receptor kinase 2 promotes high-level Hedgehog signaling by regulating the active state of Smo through kinase-dependent and kinase-independent mechanisms in Drosophila. Genes Dev..

[27] Chen Y., Jiang J. (2013). Decoding the phosphorylation code in Hedgehog signal transduction. Cell Res..

[28] Maier D., Cheng S., Faubert D., Hipfner D.R. (2014). A broadly conserved G-protein-coupled receptor kinase phosphorylation mechanism controls Drosophila smoothened activity. PLoS Genet..

[29] Zhang C., Williams E.H., Guo Y., Lum L., Beachy P.A. (2004). Extensive phosphorylation of Smoothened in Hedgehog pathway activation. Proc. Natl. Acad. Sci. USA.

[30] Jia J., Tong C., Wang B., Luo L., Jiang J. (2004). Hedgehog signaling activity of smoothened requires phosphorylation by protein kinase A and casein kinase I. Nature.

[31] Clop A., Marcq F., Takeda H., Pirottin D., Tordoir X., Bibé B., Bouix J., Caiment F., Elsen J.M., Eychenne F. (2006). A mutation creating a potential illegitimate microRNA target site in the myostatin gene affects muscularity in sheep. Nat. Genet..

[32] Stachowiak M., Szydlowski M., Flisikowski K., Flisikowska T., Bartz M., Schnieke A., Switonski M. (2014). Polymorphism in 3ʹ untranslated region of the pig PPARA gene influences its transcript level and is associated with adipose tissue accumulation. J. Anim. Sci..

[33] Akey J., Jin L., Xiong M.M. (2001). Haplotypes *vs*. single marker linkage disequilibrium tests: What do we gain?. Eur. J. Hum. Genet..

[34] Inbeagha-Awemu E.M., Kgwatalala P., Zhao X. (2008). A critical analysis of production-associated DNA polymorphisms in the genes of cattle, goat, sheep, and pig. Mamm. Genome.

[35] Weedon M.N., Lango H., Lindgren C.M., Wallace C., Evans D.M., Mangino M., Freathy R.M., Perry J.R., Stevens S., Hall A.S. (2008). Genome-wide association analysis identifies 20 loci that influence adult height. Nat. Genet..

[36] Gilbert R.P., Bailey D.R., Shannon N.H. (1993). Linear body measurements of cattle before and after 20 years of selection for postweaning gain when fed two different diets. J. Anim. Sci..

[37] Mullenbach R., Lagoda P.J., Welter C. (1989). An efficient salt-chloroform extraction of DNA from blood and tissues. Trends Genet..

[38] Nei M., Roychoudhury A.K. (1974). Sampling variance of heterozygosity and genetic distance. Genetics.

[39] Li Z., Zhang Z., He Z., Tang W., Li T., Zeng Z., He L., Shi Y. (2009). A partition-ligation-combination-subdivision EM algorithm for haplotype inference with multiallelic markers: Update of the SHEsis (http://analysis.bio-x.cn). Cell Res..

[40] Shi Y.Y., He L. (2005). SHEsis a powerful software platform for analyses of linkage disequilibrium, haplotype construction, and genetic association at polymorphism loci. Cell Res..

