# Supplementary material for: Molecular Characterization of Bovine SMO Gene and Effects of Its Genetic Variations on Body Size Traits in Qinchuan Cattle (Bos taurus)
Source: Int J Mol Sci. 2015 Jul 27;16(8):16966–80. doi: 10.3390/ijms160816966 (PMC4581179; doi:10.3390/ijms160816966)
Supplement: Supplementary file 1 [file ijms-16-16966-s001.pdf]

Supplementary Information

Table S1. Detected SNP information of the bovine SMO gene for 520 Qinchuan cattle.

Number of Cattle	Genotype							
	SNP1	SNP2	SNP3	SNP4	SNP5	SNP6	SNP7	SNP8
1	C C	T C	C T	C A	C T	T G	T C	C C
2	C C	C C	C C	C A	C C	T G	T C	C C
3	C C	T C	C C	C C	C C	T G	T C	C C
4	C C	T C	C T	C A	C T	T G	T C	C C
5	C C	T T	C C	C C	C C	T T	T T	C C
6	C C	T T	C C	C C	C C	T T	T T	C C
7	C T	C C	C C	C A	C C	G G	C C	C A
8	C C	T C	C T	C A	C T	T G	T C	C C
9	C C	T T	C C	C C	C C	T T	T T	C C
10	C C	C C	C T	A A	C T	T G	T C	C C
11	C C	C C	C T	A A	C T	T G	T C	C C
12	C C	C C	T T	A A	T T	G G	C C	C C
13	C C	T T	C C	C C	C C	T T	T T	C C
14	C T	T C	C C	C A	C C	T G	T C	C A
15	C C	T T	C C	C C	C C	T T	T T	C C
16	C C	T C	C T	C A	C T	T G	T C	C C
17	C C	T C	C T	C A	C T	T G	T C	C C
18	C T	C C	C C	C A	C C	G G	C C	C A
19	C C	T T	C C	C C	C C	T T	T T	C C
20	C C	T T	C C	C C	C C	T T	T T	C C
21	C C	T T	C C	C C	C C	T T	T T	C C
22	C C	C C	T T	A A	T T	G G	C C	C C
23	C T	T C	C C	C A	C C	T G	T C	C A
24	C C	T C	C T	C A	C T	T G	T C	C C
25	C C	C C	C T	C A	C T	G G	C C	C C
26	C C	T T	C C	C C	C C	T T	T T	C C
27	C C	T C	C T	C A	C T	T G	T C	C C
28	C C	T C	C T	C A	C T	T G	T C	C C
29	C C	T T	C C	C C	C C	T T	T T	C C
30	C C	T T	C C	C C	C C	T T	T T	C C
31	C C	T T	C C	C C	C C	T T	T T	C C
32	C C	T C	C T	C A	C T	T G	T C	C C
33	C C	T C	C C	C C	C C	T G	T C	C C
34	C C	T C	C T	C A	C T	T G	T C	C C
35	C C	C C	C C	C C	C C	G G	C C	C C
36	C C	C C	C T	A A	C T	T G	T C	C C
37	C C	T T	C C	C C	C C	T T	T T	C C
38	C C	T T	C C	C C	C C	T T	T T	C C
39	C C	T C	C C	C A	C C	T T	T T	C C
40	C C	T T	C C	C C	C C	T T	T T	C C
41	C C	T T	C C	C C	C C	T T	T T	C C
42	C C	T C	C T	C A	C T	T G	T C	C C
43	C C	T C	C T	C A	C T	T G	T C	C C

Table S1. *Cont.*

Number of Cattle	Genotype							
	SNP1	SNP2	SNP3	SNP4	SNP5	SNP6	SNP7	SNP8
44	C C	C C	C C	C A	C C	T G	T C	C C
45	C C	T T	C C	C C	C C	T T	T T	C C
46	C C	T C	C T	C A	C T	T G	T C	C C
47	C C	C C	C T	A A	C T	T G	T C	C C
48	C C	T T	C C	C C	C C	T T	T T	C C
49	C C	T C	C T	C A	C T	T G	T C	C C
50	C C	T T	C C	C C	C C	T T	T T	C C
51	C C	C C	T T	A A	T T	G G	C C	C C
52	C C	T C	C T	C A	C T	T G	T C	C C
53	C C	T C	C T	C A	C T	T G	T C	C C
54	C C	C C	C T	A A	C T	T G	T C	C C
55	C C	T T	C C	C C	C C	T T	T T	C C
56	C C	T T	C C	C C	C C	T T	T T	C C
57	C C	T C	C T	C A	C T	T G	T C	C C
58	C C	T T	C C	C C	C C	T T	T T	C C
59	C C	T C	C T	C A	C T	T G	T C	C C
60	C T	T C	C C	C A	C C	T G	T C	C A
61	C C	C C	C C	C A	C C	T G	T C	C C
62	C C	T C	C C	C C	C C	T G	T C	C C
63	C C	C C	T T	A A	T T	G G	C C	C C
64	C C	T C	C T	C A	C T	T G	T C	C C
65	C C	C C	C T	C A	C T	G G	C C	C C
66	C C	T C	C C	C C	C C	T G	T C	C C
67	C C	C C	C T	C A	C T	G G	C C	C C
68	C C	T C	C C	C A	C C	T T	T T	C C
69	C C	T C	C C	C A	C C	T T	T T	C C
70	C C	T T	C C	C C	C C	T T	T T	C C
71	C C	T T	C C	C C	C C	T T	T T	C C
72	C C	T C	C T	C A	C T	T G	T C	C C
73	C C	C C	C C	A A	C C	T T	T T	C C
74	C C	T C	C C	C A	C C	T T	T T	C C
75	C C	T C	C C	C C	C C	T G	T C	C C
76	C C	T T	C C	C C	C C	T T	T T	C C
77	C C	C C	C T	C A	C T	G G	C C	C C
78	C C	T T	C C	C C	C C	T T	T T	C C
79	C C	T C	C T	C A	C T	T G	T C	C C
80	C C	T C	C T	C A	C T	T G	T C	C C
81	C C	C C	C T	C A	C T	G G	C C	C C
82	C C	T T	C C	C C	C C	T T	T T	C C
83	C C	T C	C T	C A	C T	T G	T C	C C
84	C C	T C	C T	C A	C T	T G	T C	C C
85	C C	T C	C T	C A	C T	T G	T C	C C
86	C C	T T	C C	C C	C C	T T	T T	C C

Table S1. *Cont.*

Number of Cattle	Genotype							
	**SNP1**	**SNP2**	**SNP3**	**SNP4**	**SNP5**	**SNP6**	**SNP7**	**SNP8**
87	C T	C C	C T	A A	C T	G G	C C	C A
88	C C	T C	C T	C A	C T	T G	T C	C C
89	C C	C C	T T	A A	T T	G G	C C	C C
90	C C	T T	C C	C C	C C	T T	T T	C C
91	C C	T C	C T	C A	C T	T G	T C	C C
92	C C	T C	C T	C A	C T	T G	T C	C C
93	C C	C C	T T	A A	T T	G G	C C	C C
94	C C	T C	C T	C A	C T	T G	T C	C C
95	C C	T C	C C	C A	C C	T T	T T	C C
96	C C	T T	C C	C C	C C	T T	T T	C C
97	C C	C C	T T	A A	T T	G G	C C	C C
98	C C	T T	C C	C C	C C	T T	T T	C C
99	C C	T T	C C	C C	C C	T T	T T	C C
100	C C	T C	C T	C A	C T	T G	T C	C C
101	C C	T C	C T	C A	C T	T G	T C	C C
102	C C	T T	C C	C C	C C	T T	T T	C C
103	C C	T C	C C	C C	C C	T G	T C	C C
104	C C	T T	C C	C C	C C	T T	T T	C C
105	C C	T C	C T	C A	C T	T G	T C	C C
106	C C	T C	C T	C A	C T	T G	T C	C C
107	C T	T C	C C	C A	C C	T G	T C	C A
108	C C	T C	C C	C A	C C	T T	T T	C C
109	C C	T T	C C	C C	C C	T T	T T	C C
110	C C	T C	C C	C C	C C	T G	T C	C C
111	C C	T T	C C	C C	C C	T T	T T	C C
112	C C	T C	C T	C A	C T	T G	T C	C C
113	C C	C C	C T	C A	C T	G G	C C	C C
114	C C	T C	C T	C A	C T	T G	T C	C C
115	C C	T T	C C	C C	C C	T T	T T	C C
116	C C	C C	C T	A A	C T	T G	T C	C C
117	C C	T T	C C	C C	C C	T T	T T	C C
118	C C	T T	C C	C C	C C	T T	T T	C C
119	C T	C C	C T	A A	C T	G G	C C	C A
120	C C	C C	C T	A A	C T	T G	T C	C C
121	C C	C C	C T	C A	C T	G G	C C	C C
122	C C	T C	C T	C A	C T	T G	T C	C C
123	C C	T C	C T	C A	C T	T G	T C	C C
124	C C	T T	C C	C C	C C	T T	T T	C C
125	C C	T C	C C	C A	C C	T T	T T	C C
126	C C	T C	C C	C A	C C	T T	T T	C C
127	C C	T C	C T	C A	C T	T G	T C	C C
128	C T	C C	C T	A A	C T	G G	C C	C A
129	C C	T C	C T	C A	C T	T G	T C	C C

Table S1. *Cont.*

Number of Cattle	Genotype							
	SNP1	SNP2	SNP3	SNP4	SNP5	SNP6	SNP7	SNP8
130	C C	T T	C C	C C	C C	T T	T T	C C
131	C C	T C	C C	C C	C C	T G	T C	C C
132	C C	T C	C C	C C	C C	T G	T C	C C
133	C C	T C	C C	C C	C C	T G	T C	C C
134	C C	T C	C T	C A	C T	T G	T C	C C
135	C C	T C	C C	C A	C C	T T	T T	C C
136	C C	C C	C T	A A	C T	T G	T C	C C
137	C C	C C	T T	A A	T T	G G	C C	C C
138	C C	T C	C C	C A	C C	T T	T T	C C
139	C C	T C	C C	C C	C C	T G	T C	C C
140	C T	T C	C C	C A	C C	T G	T C	C A
141	C C	T C	C T	C A	C T	T G	T C	C C
142	C C	T T	C C	C C	C C	T T	T T	C C
143	C C	T C	C T	C A	C T	T G	T C	C C
144	C C	T C	C T	C A	C T	T G	T C	C C
145	C C	T T	C C	C C	C C	T T	T T	C C
146	C C	T T	C C	C C	C C	T T	T T	C C
147	C C	T T	C C	C C	C C	T T	T T	C C
148	C C	T C	C C	C C	C C	T G	T C	C C
149	C C	C C	C C	C A	C C	T G	T C	C C
150	C C	T C	C T	C A	C T	T G	T C	C C
151	C C	T C	C C	C C	C C	T G	T C	C C
152	C C	C C	T T	A A	T T	G G	C C	C C
153	C C	T T	C C	C C	C C	T T	T T	C C
154	C C	T C	C T	C A	C T	T G	T C	C C
155	C C	T T	C C	C C	C C	T T	T T	C C
156	C C	T C	C T	C A	C T	T G	T C	C C
157	C C	T T	C C	C C	C C	T T	T T	C C
158	C C	T C	C C	C A	C C	T T	T T	C C
159	C C	T C	C C	C A	C C	T T	T T	C C
160	C C	T C	C C	C A	C C	T T	T T	C C
161	C T	C C	C C	A A	C C	T G	T C	C A
162	C C	T C	C C	C A	C C	T T	T T	C C
163	C C	T T	C C	C C	C C	T T	T T	C C
164	C T	T C	C C	C A	C C	T G	T C	C A
165	C C	C C	C C	C A	C C	T G	T C	C C
166	C C	C C	C T	C A	C T	G G	C C	C C
167	C C	T C	C T	C A	C T	T G	T C	C C
168	C C	T C	C T	C A	C T	T G	T C	C C
169	C C	T C	C C	C A	C C	T T	T T	C C
170	C C	T T	C C	C C	C C	T T	T T	C C
171	C C	T C	C C	C A	C C	T T	T T	C C
172	C C	T T	C C	C C	C C	T T	T T	C C

Table S1. *Cont.*

Number of Cattle	Genotype							
	SNP1	SNP2	SNP3	SNP4	SNP5	SNP6	SNP7	SNP8
173	C C	T T	C C	C C	C C	T T	T T	C C
174	C C	T C	C T	C A	C T	T G	T C	C C
175	C C	T T	C C	C C	C C	T T	T T	C C
176	C C	T C	C C	C A	C C	T T	T T	C C
177	C C	T C	C T	C A	C T	T G	T C	C C
178	C C	C C	T T	A A	T T	G G	C C	C C
179	C C	T C	C T	C A	C T	T G	T C	C C
180	C C	T T	C C	C C	C C	T T	T T	C C
181	C C	T C	C T	C A	C T	T G	T C	C C
182	C C	T T	C C	C C	C C	T T	T T	C C
183	C C	T T	C C	C C	C C	T T	T T	C C
184	C C	T T	C C	C C	C C	T T	T T	C C
185	C C	T C	C C	C C	C C	T G	T C	C C
186	C C	T T	C C	C C	C C	T T	T T	C C
187	C C	T T	C C	C C	C C	T T	T T	C C
188	C C	T C	C C	C A	C C	T T	T T	C C
189	C C	T C	C T	C A	C T	T G	T C	C C
190	C T	C C	C C	C A	C C	G G	C C	C A
191	C C	T C	C C	C A	C C	T T	T T	C C
192	C C	T T	C C	C C	C C	T T	T T	C C
193	C C	T T	C C	C C	C C	T T	T T	C C
194	C T	T C	C C	C A	C C	T G	T C	C A
195	C C	T T	C C	C C	C C	T T	T T	C C
196	C C	T C	C T	C A	C T	T G	T C	C C
197	C C	T T	C C	C C	C C	T T	T T	C C
198	C C	T C	C T	C A	C T	T G	T C	C C
199	C C	T C	C T	C A	C T	T G	T C	C C
200	C C	T T	C C	C C	C C	T T	T T	C C
201	C T	C C	C T	A A	C T	G G	C C	C A
202	C C	C C	T T	A A	T T	G G	C C	C C
203	C C	T T	C C	C C	C C	T T	T T	C C
204	C C	T T	C C	C C	C C	T T	T T	C C
205	C C	T C	C T	C A	C T	T G	T C	C C
206	C C	T C	C T	C A	C T	T G	T C	C C
207	C C	T C	C T	C A	C T	T G	T C	C C
208	C C	T T	C C	C C	C C	T T	T T	C C
209	C C	T C	C C	C C	C C	T G	T C	C C
210	C C	T C	C C	C C	C C	T G	T C	C C
211	C T	C C	C C	A A	C C	T G	T C	C A
212	C C	T T	C C	C C	C C	T T	T T	C C
213	C C	T C	C T	C A	C T	T G	T C	C C
214	C C	T C	C C	C C	C C	T G	T C	C C
215	C C	C C	C T	A A	C T	T G	T C	C C

Table S1. *Cont.*

Number of Cattle	Genotype							
	SNP1	SNP2	SNP3	SNP4	SNP5	SNP6	SNP7	SNP8
216	C C	T T	C C	C C	C C	T T	T T	C C
217	C C	T C	C T	C A	C T	T G	T C	C C
218	C C	T C	C T	C A	C T	T G	T C	C C
219	C C	T C	C C	C A	C C	T T	T T	C C
220	C C	T C	C C	C A	C C	T T	T T	C C
221	C C	T C	C C	C A	C C	T T	T T	C C
222	C C	T C	C T	C A	C T	T G	T C	C C
223	C C	T C	C T	C A	C T	T G	T C	C C
224	C C	T T	C C	C C	C C	T T	T T	C C
225	C C	T T	C C	C C	C C	T T	T T	C C
226	C C	T C	C T	C A	C T	T G	T C	C C
227	C C	T C	C T	C A	C T	T G	T C	C C
228	C C	T C	C T	C A	C T	T G	T C	C C
229	C C	T C	C T	C A	C T	T G	T C	C C
230	C C	C C	T T	A A	T T	G G	C C	C C
231	C C	T C	C C	C C	C C	T G	T C	C C
232	C C	T C	C T	C A	C T	T G	T C	C C
233	C C	T T	C C	C C	C C	T T	T T	C C
234	C C	T T	C C	C C	C C	T T	T T	C C
235	C C	C C	T T	A A	T T	G G	C C	C C
236	C C	T T	C C	C C	C C	T T	T T	C C
237	C C	C C	C T	A A	C T	T G	T C	C C
238	C C	C C	T T	A A	T T	G G	C C	C C
239	C C	C C	T T	A A	T T	G G	C C	C C
240	C C	T C	C T	C A	C T	T G	T C	C C
241	C C	C C	T T	A A	T T	G G	C C	C C
242	C C	T C	C C	C C	C C	T G	T C	C C
243	C C	T T	C C	C C	C C	T T	T T	C C
244	C C	T C	C T	C A	C T	T G	T C	C C
245	C C	T T	C C	C C	C C	T T	T T	C C
246	C C	T T	C C	C C	C C	T T	T T	C C
247	C C	T T	C C	C C	C C	T T	T T	C C
248	C C	T T	C C	C C	C C	T T	T T	C C
249	C C	T C	C T	C A	C T	T G	T C	C C
250	C C	T C	C C	C A	C C	T T	T T	C C
251	C T	T C	C C	C A	C C	T G	T C	C A
252	C T	C C	C T	A A	C T	G G	C C	C A
253	C C	T C	C T	C A	C T	T G	T C	C C
254	C C	T T	C C	C C	C C	T T	T T	C C
255	C C	T T	C C	C C	C C	T T	T T	C C
256	C C	T C	C T	C A	C T	T G	T C	C C
257	C C	T C	C T	C A	C T	T G	T C	C C
258	C C	T C	C T	C A	C T	T G	T C	C C

Table S1. *Cont.*

Number of Cattle	Genotype							
	SNP1	SNP2	SNP3	SNP4	SNP5	SNP6	SNP7	SNP8
259	C C	T C	C C	C C	C C	T G	T C	C C
260	C C	T T	C C	C C	C C	T T	T T	C C
261	C C	C C	C T	A A	C T	T G	T C	C C
262	C C	T C	C C	C A	C C	T T	T T	C C
263	C C	C C	C T	C A	C T	G G	C C	C C
264	C C	T C	C C	C C	C C	T G	T C	C C
265	C C	T C	C T	C A	C T	T G	T C	C C
266	C C	T C	C T	C A	C T	T G	T C	C C
267	C C	T C	C T	C A	C T	T G	T C	C C
268	C C	T T	C C	C C	C C	T T	T T	C C
269	C T	T C	C C	C A	C C	T G	T C	C A
270	C C	T C	C T	C A	C T	T G	T C	C C
271	C C	T C	C C	C A	C C	T T	T T	C C
272	C C	T T	C C	C C	C C	T T	T T	C C
273	C C	T C	C C	C A	C C	T T	T T	C C
274	C C	C C	C T	A A	C T	T G	T C	C C
275	C C	T T	C C	C C	C C	T T	T T	C C
276	C C	C C	C T	C A	C T	G G	C C	C C
277	C C	T T	C C	C C	C C	T T	T T	C C
278	C C	T C	C T	C A	C T	T G	T C	C C
279	C C	T T	C C	C C	C C	T T	T T	C C
280	C C	T C	C T	C A	C T	T G	T C	C C
281	C C	T C	C C	C A	C C	T T	T T	C C
282	C C	T T	C C	C C	C C	T T	T T	C C
283	C C	T C	C C	C C	C C	T G	T C	C C
284	C C	C C	C C	C A	C C	T G	T C	C C
285	C C	T T	C C	C C	C C	T T	T T	C C
286	C C	T T	C C	C C	C C	T T	T T	C C
287	C C	T C	C C	C A	C C	T T	T T	C C
288	C C	T T	C C	C C	C C	T T	T T	C C
289	C C	C C	T T	A A	T T	G G	C C	C C
290	C C	C C	C C	A A	C C	T T	T T	C C
291	C C	T C	C C	C C	C C	T G	T C	C C
292	C C	C C	T T	A A	T T	G G	C C	C C
293	C C	C C	T T	A A	T T	G G	C C	C C
294	C C	T C	C T	C A	C T	T G	T C	C C
295	C C	T C	C C	C C	C C	T G	T C	C C
296	C C	T C	C C	C A	C C	T T	T T	C C
297	C C	T C	C T	C A	C T	T G	T C	C C
298	C C	T C	C T	C A	C T	T G	T C	C C
299	C T	T C	C C	C A	C C	T G	T C	C A
300	C C	T C	C C	C C	C C	T G	T C	C C
301	C C	C C	C T	A A	C T	T G	T C	C C

Table S1. *Cont.*

Number of Cattle	Genotype							
	**SNP1**	**SNP2**	**SNP3**	**SNP4**	**SNP5**	**SNP6**	**SNP7**	**SNP8**
302	C C	T T	C C	C C	C C	T T	T T	C C
303	C C	T C	C T	C A	C T	T G	T C	C C
304	C C	T C	C T	C A	C T	T G	T C	C C
305	C C	T C	C T	C A	C T	T G	T C	C C
306	C C	T T	C C	C C	C C	T T	T T	C C
307	C C	T C	C T	C A	C T	T G	T C	C C
308	C C	T C	C C	C C	C C	T G	T C	C C
309	C C	T C	C T	C A	C T	T G	T C	C C
310	C C	T C	C T	C A	C T	T G	T C	C C
311	C C	T C	C T	C A	C T	T G	T C	C C
312	C C	T T	C C	C C	C C	T T	T T	C C
313	C C	C C	C C	C C	C C	G G	C C	C C
314	C C	T T	C C	C C	C C	T T	T T	C C
315	C C	C C	C T	C A	C T	G G	C C	C C
316	C C	T C	C T	C A	C T	T G	T C	C C
317	C C	T C	C T	C A	C T	T G	T C	C C
318	C C	T C	C C	C A	C C	T T	T T	C C
319	C C	T T	C C	C C	C C	T T	T T	C C
320	C C	T T	C C	C C	C C	T T	T T	C C
321	C C	T C	C C	C C	C C	T G	T C	C C
322	C C	T C	C T	C A	C T	T G	T C	C C
323	C C	T C	C C	C A	C C	T T	T T	C C
324	C C	T C	C C	C A	C C	T T	T T	C C
325	C C	T T	C C	C C	C C	T T	T T	C C
326	C C	T T	C C	C C	C C	T T	T T	C C
327	C C	T T	C C	C C	C C	T T	T T	C C
328	C C	T C	C C	C C	C C	T G	T C	C C
329	C C	T C	C C	C A	C C	T T	T T	C C
330	C C	T C	C C	C A	C C	T T	T T	C C
331	C C	C C	T T	A A	T T	G G	C C	C C
332	C C	T C	C C	C A	C C	T T	T T	C C
333	C C	T C	C T	C A	C T	T G	T C	C C
334	C C	T C	C C	C C	C C	T G	T C	C C
335	C C	T C	C C	C C	C C	T G	T C	C C
336	C C	T T	C C	C C	C C	T T	T T	C C
337	C C	C C	C T	C A	C T	G G	C C	C C
338	C C	T C	C T	C A	C T	T G	T C	C C
339	C T	T C	C C	C A	C C	T G	T C	C A
340	C C	T C	C C	C C	C C	T G	T C	C C
341	C T	T C	C C	C A	C C	T G	T C	C A
342	C C	T T	C C	C C	C C	T T	T T	C C
343	C C	T C	C C	C A	C C	T T	T T	C C
344	C C	T C	C T	C A	C T	T G	T C	C C

Table S1. *Cont.*

Number of Cattle	Genotype							
	SNP1	SNP2	SNP3	SNP4	SNP5	SNP6	SNP7	SNP8
345	C C	T T	C C	C C	C C	T T	T T	C C
346	C C	T T	C C	C C	C C	T T	T T	C C
347	C C	T C	C T	C A	C T	T G	T C	C C
348	C C	T T	C C	C C	C C	T T	T T	C C
349	C C	T C	C T	C A	C T	T G	T C	C C
350	C C	T C	C C	C C	C C	T G	T C	C C
351	C C	T T	C C	C C	C C	T T	T T	C C
352	C C	T T	C C	C C	C C	T T	T T	C C
353	C C	T C	C C	C A	C C	T T	T T	C C
354	C C	T C	C C	C C	C C	T G	T C	C C
355	C T	C C	C T	A A	C T	G G	C C	C A
356	C C	T C	C C	C A	C C	T T	T T	C C
357	C C	T T	C C	C C	C C	T T	T T	C C
358	C C	C C	C T	A A	C T	T G	T C	C C
359	C C	T C	C T	C A	C T	T G	T C	C C
360	C C	T C	C T	C A	C T	T G	T C	C C
361	C C	T C	C C	C C	C C	T G	T C	C C
362	C C	T C	C T	C A	C T	T G	T C	C C
363	C C	T C	C C	C A	C C	T T	T T	C C
364	C C	C C	C T	C A	C T	G G	C C	C C
365	C C	T C	C C	C C	C C	T G	T C	C C
366	C C	T C	C C	C A	C C	T T	T T	C C
367	C C	T C	C T	C A	C T	T G	T C	C C
368	C C	T T	C C	C C	C C	T T	T T	C C
369	C C	T C	C T	C A	C T	T G	T C	C C
370	C C	T C	C T	C A	C T	T G	T C	C C
371	C T	T C	C C	C A	C C	T G	T C	C A
372	C C	T C	C T	C A	C T	T G	T C	C C
373	C C	T C	C T	C A	C T	T G	T C	C C
374	C C	C C	T T	A A	T T	G G	C C	C C
375	C T	C C	C T	A A	C T	G G	C C	C A
376	C C	T T	C C	C C	C C	T T	T T	C C
377	C C	T C	C T	C A	C T	T G	T C	C C
378	C C	T T	C C	C C	C C	T T	T T	C C
379	C C	C C	C C	C A	C C	T G	T C	C C
380	C C	C C	C T	A A	C T	T G	T C	C C
381	C C	C C	C T	C A	C T	G G	C C	C C
382	C C	T C	C T	C A	C T	T G	T C	C C
383	C C	T T	C C	C C	C C	T T	T T	C C
384	C C	C C	C T	C A	C T	G G	C C	C C
385	C C	T C	C T	C A	C T	T G	T C	C C
386	C C	T T	C C	C C	C C	T T	T T	C C
387	C C	T T	C C	C C	C C	T T	T T	C C

Table S1. *Cont.*

Number of Cattle	Genotype							
	SNP1	SNP2	SNP3	SNP4	SNP5	SNP6	SNP7	SNP8
388	C C	T C	C T	C A	C T	T G	T C	C C
389	C C	T C	C C	C C	C C	T G	T C	C C
390	C C	T C	C C	C A	C C	T T	T T	C C
391	C C	T C	C T	C A	C T	T G	T C	C C
392	C C	T T	C C	C C	C C	T T	T T	C C
393	C C	T T	C C	C C	C C	T T	T T	C C
394	C T	C C	C T	A A	C T	G G	C C	C A
395	C C	T C	C C	C A	C C	T T	T T	C C
396	C C	T C	C T	C A	C T	T G	T C	C C
397	C C	T C	C T	C A	C T	T G	T C	C C
398	C C	T C	C C	C C	C C	T G	T C	C C
399	C C	T T	C C	C C	C C	T T	T T	C C
400	C C	T C	C T	C A	C T	T G	T C	C C
401	C C	T C	C T	C A	C T	T G	T C	C C
402	C C	T C	C C	C C	C C	T G	T C	C C
403	C C	T T	C C	C C	C C	T T	T T	C C
404	C C	T T	C C	C C	C C	T T	T T	C C
405	C C	T C	C T	C A	C T	T G	T C	C C
406	C C	T T	C C	C C	C C	T T	T T	C C
407	C C	T T	C C	C C	C C	T T	T T	C C
408	C C	T C	C C	C C	C C	T G	T C	C C
409	C C	T C	C T	C A	C T	T G	T C	C C
410	C C	T T	C C	C C	C C	T T	T T	C C
411	C C	T T	C C	C C	C C	T T	T T	C C
412	C C	T C	C C	C A	C C	T T	T T	C C
413	C C	T C	C T	C A	C T	T G	T C	C C
414	C C	T C	C C	C A	C C	T T	T T	C C
415	C T	T C	C C	C A	C C	T G	T C	C A
416	C C	T T	C C	C C	C C	T T	T T	C C
417	C C	T C	C C	C C	C C	T G	T C	C C
418	C C	T T	C C	C C	C C	T T	T T	C C
419	C C	T C	C C	C C	C C	T G	T C	C C
420	C C	T T	C C	C C	C C	T T	T T	C C
421	C C	T T	C C	C C	C C	T T	T T	C C
422	C C	T C	C T	C A	C T	T G	T C	C C
423	C T	C C	C T	A A	C T	G G	C C	C A
424	C C	T C	C T	C A	C T	T G	T C	C C
425	C C	T T	C C	C C	C C	T T	T T	C C
426	C C	T C	C T	C A	C T	T G	T C	C C
427	C C	T C	C T	C A	C T	T G	T C	C C
428	C C	T C	C C	C C	C C	T G	T C	C C
429	C T	T C	C C	C A	C C	T G	T C	C A
430	C C	T C	C C	C C	C C	T G	T C	C C

Table S1. *Cont.*

Number of Cattle	Genotype							
	SNP1	SNP2	SNP3	SNP4	SNP5	SNP6	SNP7	SNP8
431	C C	T T	C C	C C	C C	T T	T T	C C
432	C C	T C	C C	C C	C C	T G	T C	C C
433	C C	C C	C T	C A	C T	G G	C C	C C
434	C C	T C	C C	C C	C C	T G	T C	C C
435	C C	T C	C T	C A	C T	T G	T C	C C
436	C C	C C	C T	C A	C T	G G	C C	C C
437	C C	T C	C C	C A	C C	T T	T T	C C
438	C C	T C	C C	C A	C C	T T	T T	C C
439	C C	T T	C C	C C	C C	T T	T T	C C
440	C C	T C	C C	C C	C C	T G	T C	C C
441	C C	T C	C C	C A	C C	T T	T T	C C
442	C C	T T	C C	C C	C C	T T	T T	C C
443	C C	T C	C C	C C	C C	T G	T C	C C
444	C C	T T	C C	C C	C C	T T	T T	C C
445	C C	T T	C C	C C	C C	T T	T T	C C
446	C C	T C	C C	C C	C C	T G	T C	C C
447	C C	T C	C C	C C	C C	T G	T C	C C
448	C C	T T	C C	C C	C C	T T	T T	C C
449	C C	C C	T T	A A	T T	G G	C C	C C
450	C C	C C	C T	A A	C T	T G	T C	C C
451	C C	T C	C T	C A	C T	T G	T C	C C
452	C C	T C	C C	C C	C C	T G	T C	C C
453	C C	C C	C T	C A	C T	G G	C C	C C
454	C C	T C	C T	C A	C T	T G	T C	C C
455	C C	T T	C C	C C	C C	T T	T T	C C
456	C C	T C	C C	C C	C C	T G	T C	C C
457	C C	T C	C T	C A	C T	T G	T C	C C
458	C T	C C	C C	C A	C C	G G	C C	C A
459	C C	T C	C T	C A	C T	T G	T C	C C
460	C C	T T	C C	C C	C C	T T	T T	C C
461	C C	T T	C C	C C	C C	T T	T T	C C
462	C C	C C	C T	A A	C T	T G	T C	C C
463	C C	T C	C C	C A	C C	T T	T T	C C
464	C C	T C	C C	C C	C C	T G	T C	C C
465	C C	C C	T T	A A	T T	G G	C C	C C
466	C C	T C	C T	C A	C T	T G	T C	C C
467	C C	T T	C C	C C	C C	T T	T T	C C
468	C C	T C	C T	C A	C T	T G	T C	C C
469	C C	T T	C C	C C	C C	T T	T T	C C
470	C C	T C	C C	C A	C C	T T	T T	C C
471	C C	T C	C T	C A	C T	T G	T C	C C
472	C C	T C	C C	C A	C C	T T	T T	C C
473	C C	T C	C T	C A	C T	T G	T C	C C

Table S1. *Cont.*

Number of Cattle	Genotype							
	SNP1	SNP2	SNP3	SNP4	SNP5	SNP6	SNP7	SNP8
474	C C	T C	C C	C C	C C	T G	T C	C C
475	C C	C C	C C	C A	C C	T G	T C	C C
476	C C	T C	C C	C C	C C	T G	T C	C C
477	C C	T T	C C	C C	C C	T T	T T	C C
478	C C	T C	C T	C A	C T	T G	T C	C C
479	C C	T T	C C	C C	C C	T T	T T	C C
480	C C	T T	C C	C C	C C	T T	T T	C C
481	C C	T C	C C	C A	C C	T T	T T	C C
482	C C	T T	C C	C C	C C	T T	T T	C C
483	C T	T C	C C	C A	C C	T G	T C	C A
484	C C	T C	C T	C A	C T	T G	T C	C C
485	C C	T T	C C	C C	C C	T T	T T	C C
486	C T	C C	C C	A A	C C	T G	T C	C A
487	C C	T T	C C	C C	C C	T T	T T	C C
488	C C	T T	C C	C C	C C	T T	T T	C C
489	C C	C C	T T	A A	T T	G G	C C	C C
490	C C	T C	C T	C A	C T	T G	T C	C C
491	C C	T C	C C	C A	C C	T T	T T	C C
492	C C	T C	C T	C A	C T	T G	T C	C C
493	C C	C C	T T	A A	T T	G G	C C	C C
494	C C	T T	C C	C C	C C	T T	T T	C C
495	C C	T T	C C	C C	C C	T T	T T	C C
496	C C	C C	C T	C A	C T	G G	C C	C C
497	C C	T C	C C	C C	C C	T G	T C	C C
498	C C	T T	C C	C C	C C	T T	T T	C C
499	C C	C C	C T	C A	C T	G G	C C	C C
500	C C	T T	C C	C C	C C	T T	T T	C C
501	C C	T T	C C	C C	C C	T T	T T	C C
502	C C	T C	C C	C C	C C	T G	T C	C C
503	C C	T T	C C	C C	C C	T T	T T	C C
504	C C	T T	C C	C C	C C	T T	T T	C C
505	C C	T C	C C	C A	C C	T T	T T	C C
506	C C	C C	T T	A A	T T	G G	C C	C C
507	C C	T T	C C	C C	C C	T T	T T	C C
508	C C	T C	C T	C A	C T	T G	T C	C C
509	C C	C C	T T	A A	T T	G G	C C	C C
510	C C	T C	C C	C C	C C	T G	T C	C C
511	C C	T T	C C	C C	C C	T T	T T	C C
512	C C	T T	C C	C C	C C	T T	T T	C C
513	C C	T T	C C	C C	C C	T T	T T	C C
514	C C	T C	C C	C C	C C	T G	T C	C C
515	C C	T T	C C	C C	C C	T T	T T	C C
516	C C	T T	C C	C C	C C	T T	T T	C C

Table S1. *Cont.*

Number of Cattle	Genotype							
	SNP1	SNP2	SNP3	SNP4	SNP5	SNP6	SNP7	SNP8
517	C C	T T	C C	C C	C C	T T	T T	C C
518	C C	T C	C T	C A	C T	T G	T C	C C
519	C T	C C	C T	A A	C T	G G	C C	C A
520	C C	T C	C C	C C	C C	T G	T C	C C

Table S2. Primers for detecting and genotyping SNPs of the bovine SMO gene by PCR direct sequencing.

Functions	Primer Sequences (5′-3′)	Location	Annealing Temperature (°C)
SNP Detecting	F: AAATGCTCGGCTGAGAAATG<br>R: AACCCGTTTGATTCCATCTC	Exon 2	55.8
SNP Detecting	F: CCTGCTGCTTTCCGTTGTAT<br>R: GAACTCCCCACCCCTCTAAT	Exon 3	57.8
SNP Detecting	F: GTGGCAGAACAGCCCTAACC<br>R: CTGAATCCCACGCTGAAGTC	Exon 4	57.8
SNP Detecting	F: TAGAGCCTGGACCGTTCACA<br>R: TAGCCCACAAAACAGATGCC	Exon 5	57.8
SNP Detecting	F: CCTCACTGTGGCAATCCTCG<br>R: CTCCTGGCTCCTGCTTACAC	Exon 6	56.5
SNP Detecting	F: CCAGTGTCTCCAGTTCCTCC<br>R: CTACTACCAGCATCACCGCC	Exon 7	57.8
SNP Detecting	F: GAACACCTCCTCCCTACCG<br>R: AGCACCCAAAGCAAGACAG	Exon 8	57.8
SNP Detecting	F: GGAGGGAATGGATTTACTGG<br>R: GAAGGGAACTGAGAATGGGC	Exon 10	58
SNP Detecting	F: CACAGAGCTTAGAGTCCCAG<br>R: AAGCCTCGGAACGGTATTTGT	Exon 12	56.6
SNP Detecting	F: CTCATGGATGCAGACTCCGAC<br>R: GTGGGTTACTGGCCTACGG	3′ UTR	67
Genotyping	F: TGGCTGGTTGAGGCAGAGAT<br>R: GTGGGTTACTGGCCTACGG	Exon 12 and 3′ UTR	63.1

F: Forward primer; R: Reverse primer.
